# Impact of herd mobility on brucellosis seroprevalence and spread risk resulting from cross‐border transhumance

**DOI:** 10.1002/vms3.1446

**Published:** 2024-04-09

**Authors:** Wilfried Délé Oyetola, Samba Daou, Bassirou Bonfoh, Rianatou Bada Alambedji

**Affiliations:** ^1^ Ecole Inter‐Etats des Sciences et Médecine Vétérinaires Dakar Senegal; ^2^ Centre Suisse de Recherche Scientifique en Côte d'Ivoire Abidjan Ivory Coast

**Keywords:** brucellosis, cattle, Mali, sedentary, transhumance

## Abstract

**Background:**

Cross‐border livestock mobility through transhumance is mainly practiced in West African countries for seasonal access to resources and market. Cross‐border herds are involved in the dynamic of transboundary animal diseases among them brucellosis taken as model. Brucellosis is a zoonotic disease causing abortion.

**Objectives:**

This study explores the seroprevalence of brucellosis according to mobility and infection spread between Mali and Côte d'Ivoire in the context of seasonal cross‐border transhumance.

**Methods:**

From February to April 2021, a transversal serological survey of brucellosis was conducted on 521 cattle from 111 transhumant herds and 283 cattle from 59 sedentary herds, all from Mali.

**Results:**

The global individual seroprevalence for *Brucella* spp. in transhumant and sedentary cattle from Mali was 8.2% (95% CI = 6.0–10.5). At herd level, seroprevalence was 21.2% with a significant variation between transhumant (11.7%) and sedentary (39.0%) herds. For herds in transhumance, cattle seropositivity was associated with a previous infection suspected by herdsmen odds ratio (OR = 4.4; 95% CI = 1.1–18.1) and unknown abortion aetiology (OR = 4.3; 95% CI = 1.0–17.3). The departure region (coming from Sikasso) and previous brucellosis infection or unexplained abortion could be used to predict *Brucella* infection in transhumant herds with a probability of around 60%. The risk of brucellosis introduction in host regions was high despite the individual animal seroprevalence of 3.6% and a low sale rate in transhumant cattle.

**Conclusions:**

The findings suggest that testing transhumant during border control and survey of cattle markets and sales could improve risk control of the spread of disease at regional scale.

## Graphical abstract

This study aims to explore the seroprevalence of brucellosis according to mobility, and the infection spread between Mali and Côte d'Ivoire in the context of cross‐border seasonal transhumance. Herd‐level brucellosis seroprevalence was significantly higher in sedentary herds (39.0% of herds) than in transhumant herds (11.7%) in Mali. The risk of brucellosis spreading to host regions was between 70% and 90%, and resulted from 3.6% individual seroprevalence in transhumant cattle, with 0.3%–0.6% of transhumant cattle sold.

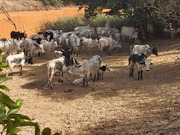

## 1 INTRODUCTION

Transhumance is one of the non‐commercial mobility of livestock (Bassett & Turner, 2007). It is a livestock production system based on the seasonal movement of herds mainly in search of water and pasture, and which return to a fixed base, each year. On the one hand, cross‐border transhumance is mutually beneficial for Sahelian countries, departing areas and Coastal countries that are hosting areas (Thebaud, 2017). On the other hand, it leads to the transmission of transboundary animal diseases such as brucellosis (Apolloni et al., 2019; Bruce et al., 2019).

Brucellosis is caused by different species of *Brucella* bacteria, whose infection leads to abortion and loss of milk production in livestock (Bruce et al., 2019), and abortion and undulating fever in human (Khan et al., 2001). The sources of infection are infected animals, more specifically their milk and abortive materials, and infected environment such as pastoral fields. In the absence of hygroma due to chronic infection in the animal, the diagnosis is based on serological tests such as Rose Bengal Test (RBT) or bacterial identification in the laboratory.

According to statistic from FAO (FAOSTAT), Mali is the second biggest pastoral country in West Africa, with 15.2% of domestic ruminants in the region (Food and Agriculture Organization [FAO], 2020). Although 15% of herds are mobile, nomadic and mostly transhumance production system concerns 70%–90% of national livestock (Ham et al., 2012). One of the main transit and host countries of transhumance from Mali is Côte d'Ivoire. The two countries share a border of 532 km but do not have any bilateral agreement concerning cross‐border transhumance. However, as member countries of the Economic Community of West African States (ECOWAS), the International Certificate of Transhumance (ICT) is used to control transhumance and provide data about animal health and their regulatory vaccines (Economic Community of West African States [ECOWAS], 1998). The list of diseases monitored with ICT does not include a lot of priority zoonoses such as brucellosis.

The cross‐border mobility, including livestock trade, and weaknesses in border control such as no laboratory diagnosis or deviation from the official transhumance routes by pastoralists, constitute determinants of brucellosis spread between Mali and Côte d'Ivoire (Oyetola et al., 2021). Indeed, the evidence of risk for disease spread from cross‐border livestock trade has been demonstrated (Dean et al., 2013). Little is known on *Brucella* contamination mechanisms and dynamics through transhumance activities, despite a published example of disease introduction caused by affected cattle coming from a neighbouring country for pasture (Kouba, 2003). However, contact with pastoralists was identified as a risk factor for sedentary cattle infection (Kanouté et al., 2017). Thus, we can assume that herds coming from the infected country would spread brucellosis during their transhumance, and a correlation of infection would exist between transhumant herds and those sedentary in the origin country.

Brucellosis is commonly present in Mali, both in domestic ruminants involved in transhumance and in humans. The causal specie for livestock brucellosis in Mali is *Brucella abortus* (Tounkara et al., 1994). According to the latest scientific paper published, the brucellosis seroprevalence in cattle is 23.3% in Mali (Tounkara et al., 1994) and 4.8% (95% IC = 3.1–6.5) in Cote d'Ivoire (Oyetola et al., 2022). However, in small ruminants, seroprevalence is 4.1% (95% CI = 2.8–5.6) without significant difference between husbandry systems (Traoré et al., 2021).

The lack of knowledge about the difference of *Brucella* infection regarding livestock mobility constitutes a gap to address risk‐based surveillance and prioritize the way of disease control. This study aims to (i) explore the link between the exposition to *Brucella spp*. and mobility by comparing seroprevalence between transhumance herds and sedentary herds from Mali and (ii) determine the risk of disease spread from transhumant herds in the hosting country.

## 2 MATERIALS AND METHODS

### 2.1 Study area

**FIGURE 1:**
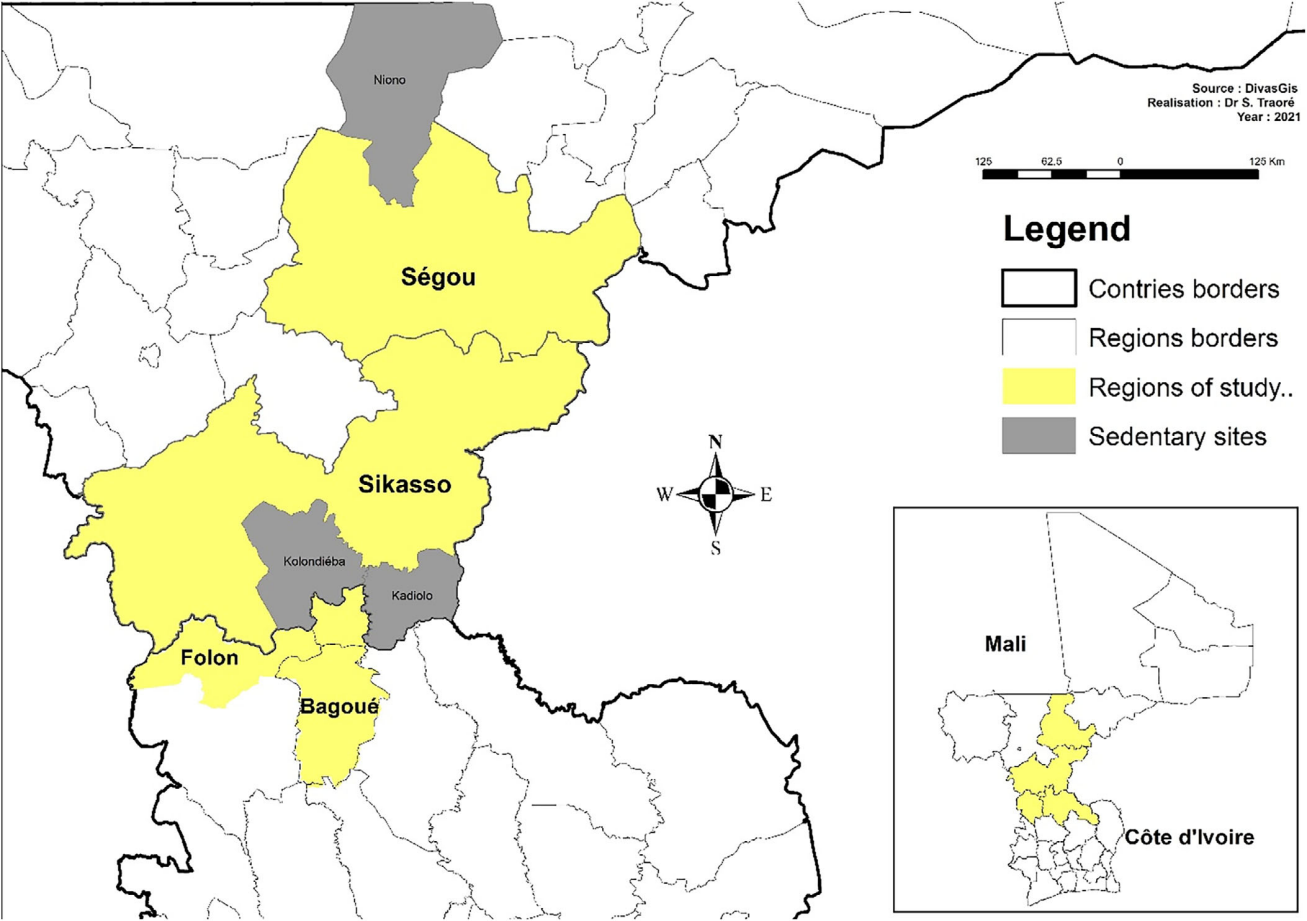
Locations of study areas in Côte d'Ivoire and Mali. Transhumant and sedentary herds were all sampled in borders regions in Côte d'Ivoire and Mali, respectively.

This cross‐sectional study was conducted during pastoral transhumance in the transit/host and departure areas of pastoral herds from Mali. The study areas were the Folon and Bagoue neighbouring regions in Côte d'Ivoire and Segou and Sikasso neighbouring regions in Mali (Figure 1).

During the last transhumance season from January to May 2020, 33,180 and 20,959 cattle from Mali entered through the region of Bagoue and Folon, respectively (Comité inter‐État de lutte contre la sécheresse au Sahel [CILSS], 2020). The regions of Folon and Bagoue located in the north of Côte d'Ivoire bordering the region of Sikasso are the main entry points for animals coming on foot from Mali. Folon and Bagoue regions have a border control post managed directly by the head of departmental veterinary health services and located in the departments of Minignan and Tingrela, respectively. To better cover the border areas, each departmental veterinary service has at least two entry posts. The climate in both regions is Sudanian tropical type, and the landscape is covered by savannahs, characterized by large areas of grassland and sparse trees vegetation. These regions have agropastoral vocation; the main products are cotton and cashew nuts. The cotton harvest lasting from October to January (Ochou et al., 2005) is decisive for the beginning of the transhumance because of the large agricultural areas that cover many pastoral tracks. Local cattle are used like oxen for work in the cotton culture, for meat production and to a lesser extent for milk production.

The regions of Sikasso and Segou were chosen because they were the main regions of origin of transhumant herds into Côte d'Ivoire in 2019, with 32,634 and 3393 cattle controlled in Malian border control posts, respectively (Direction Nationale des Services Vétérinaires [DNSV], 2020). The regions of Sikasso and Segou constitute 15.9% and 11.3% of Mali's cattle population, respectively (Direction Nationale des Productions et des Industries Animales [DNPIA], 2016). Sikasso region, like the north of Côte d'Ivoire, has a tropical Sudanian climate with rainy season from May to October and dry season from November to April, whereas Segou region located in central Mali has a tropical climate of the Sudano‐Sahelian type with a short rainy season from June to September, followed by a long dry season from October to May or June.

### 2.2 Sample size

A public database of transhumance does not exist, and official control registration did not mention a phone number of pastoralists in transhumance. Therefore, to define the sampling herd, we used following formula including the design effect of a cluster sample to a confidence level of 95% (Dohoo et al., 2014):

(1) $$n\;=\frac{z^{2}\;\times \;p\;\left(1-p\right)}{d^{2}}\;\times \mathrm{DE},$$

and

(2) $$\mathrm{DE}\;=\;\left(1+\rho \times \left(m-1\right)\right),$$

where *n* is the sample size of herd; *p* is the expected seroprevalence; *d* is the accepted precision; *z* = 1.96; DE is the design effect due to cluster; *m* is the number of cattle per herd; and *ρ* is the intra cluster correlation coefficient.

The sample size was based on expected brucellosis seroprevalence of 23.3% in cattle in Mali in 1991 (Tounkara et al., 1994) because all cattle in the study were from Mali. Although this publication is old, we assumed that the lacks of brucellosis control programmes in the country lead to the maintenance of this infection level (Oyetola et al., 2021) in both transhumant and sedentary cattle herds. In each herd, we decided to sample five cattle at least 6 months old. The intra‐herd correlation coefficient considered for the diagnosis of *B. abortus* infection in cattle using Rose Bengal plate test is *ρ* = 0.1 (Otte & Gumm, 1997). We accepted a precision *d* = 7.3%. We calculate that the correct sample size for this study was 900 cattle from 180 herds. We chose to sample a ratio of two transhumant herds per one sedentary herd, because the number of transhumant cattle is greater than that of sedentary cattle. Therefore, we should sample 120 transhumant herds in Côte d'Ivoire (Folon and Bagoue) and 60 sedentary herds in Mali (Segou and Sikasso).

### 2.3 Sample herd and data collection

Data collection was done by three survey teams; one in the region of Folon from February to April 2021 and Segou in May 2021, another one in the region of Bagoue from January to May 2021, and the third in the region of Sikasso in May 2021. Each team is composed of three or four persons: a staff member from the local veterinary station, a support agent, a nurse (non‐permanent) and a veterinarian or veterinary student.

In the region of Folon and Bagoue, we used local veterinary health services, and a network composed by mentors of pastoralist and young people of villages to give an alert when a herd in transhumance was observed in their locality. In addition, both teams visited two water points located on the transhumance track weekly. The survey team of the respective region visited the herd and presented the study to the main herdsman. The inclusion criteria were moving from Mali to Côte d'Ivoire for transhumance, accepting to attend the study by giving written consent and having cattle in the herd. One herd not included in this study was composed only by sheep, and samples were seronegative to brucellosis. The criteria of exclusion of herds in this study were obtaining a single sample that can be used in the laboratory. A member of the team eventually supports by a translator conducted interview based on a structured questionnaire in French and Fulani or Bambara language with the main herdsman of the sampled herd. Data collected on transhumant herds were the composition of herds (species and number), the locality of departure in Mali, the entry locality in Côte d'Ivoire and the final destination, the history of brucellosis (animal cases and management, observed signs) and number of cattle sale.

In the regions of Segou and Sikasso, herds were chosen randomly from a list of herds given by the local veterinary health services or private veterinarians with the veterinary mandate from the animal health ministry. The criteria of no inclusion were going history of transhumance or move out of the region during the last 5 years and refusal to participate to this study by the breeder. From each sedentary herd, information on their composition (species and number), history of contact with transhumant herds and history of brucellosis were collected.

The selection of animals was partly random according to the capacity to capture the animal, as, if possible, the main breeding male was chosen among those to be sampled from the herd. Cattle were unvaccinated against brucellosis. Data collected on each animal sampled from transhumant and sedentary herds were age, sex and breed (*Bos taurus*, *Bos indicus* or their crossing). The age was determined from the dentition of the animal (Poivey et al., 1981) and the herdsmen knowledge. A biological sample of 5 mL of blood from jugular vein was taken into a plain tube by a veterinarian following standard operating procedure for collection of blood sample in cattle (Macdonald Campus Farm Cattle Complex, 2018).

In total, 804 sera were tested from 111 transhumant herds and 59 sedentary herds with 521 and 283 sera, respectively. Most of transhumant herds (81%) were constituted of cattle only, and all the rest (19%) were cattle mixed with sheep. The average herd size was 87 cattle with a minimum of 25 and a maximum of 260. Transhumant herds were sampled in Folon (40.5%, *N* = 45) and Bagoue (59.5%, *N* = 66) regions located in Côte d'Ivoire, and sedentary herds in Segou (42.4%, *N* = 25) and Sikasso (57.6%, *N* = 34) regions of Mali.

### 2.4 Laboratory analyses

Blood samples were centrifuged in the laboratory of the local hospital in each region, at 3000 revolutions per minute for 10 min. The sera obtained was removed into a cryotube and stored in a freezer at −20°C, until expedition to the veterinary laboratory for diagnosis. The serological test performed for screen exposition to *Brucella spp* was RBT with relative sensitivity and specificity estimate from 89.6% and 84.5% (Getachew et al., 2016) to 98.9% and 100% (Gusi et al., 2019), respectively. Using micropipette, 30 µL of the Rose Bengal antigen (*B. abortus* strain S 99) and 30 µL of serum were placed alongside on the white plate, mixed and gently rocked for 4 min. The result was observed immediately; a positive reaction occurs with the presence of agglutination indicates a brucellosis infection (Pfukenyi et al., 2020).

### 2.5 Data analyses

Descriptive data of herds in transhumance were analysed to characterize them by average herd size, species composition, proportion of depart and host regions. A herd was considered positive if at least one bovine in that herd was positive. Age of sampled cattle was classified in three categories (6 months to 4 years, from 5 to 8 years and 9 years and above) used in a study of Kanoute at al. (2017). The analyses were made using pivot table in Microsoft Excel 2016.

Seroprevalence with confidence interval was calculated at an individual and herd level according to localities, sex, breed and age categories for transhumant and sedentary cattle, with the following formulas (Dohoo et al., 2014), (Bennett et al., 1991),:

(3) $$p\;=\frac{n_{p}}{n_{t}}\;$$

and

(4) $$\mathrm{CI}\;=\;p\pm z\sqrt{\frac{\mathrm{DE}\times \;p\times \left(1-p\right)}{n_{t}}}$$

where *p* is the sample seroprevalence; *n_p_* is the number of positive cases; *n_t_* is the number of tested cases; CI is the confidence interval; *z* = 1.96; DE is the design effect due to clustering sample, DE = 1.4 obtained in Formula (2).

The package Rcmdr (Fox & Bouchet‐Valat, 2020) in R software version 4.1.1 (R Core Team, 2021) was used to perform statistical analysis. Comparisons between seroprevalences were done using Fisher's exact test to calculate *p*‐value, odds ratio (OR) and its confidence interval, together. Difference between seroprevalences was declared significant for *p*‐values ≤0.05, and the associations were admitted when the confidence interval of OR did not include in 1. Concordance between previous infection and unexplained abortion, both in herds, was tested using kappa coefficient (Dohoo et al., 2014).

Multivariate logistic regression model in R was used to obtain a model for predicting current brucellosis infection of herds in transhumance. Variables tested were history of brucellosis, history of abortion, herd size, herd composition (mixed or not), departure and host regions in Mali and Côte d'Ivoire respectively, and management of brucellosis cases. A stepwise process was engaged to choose the best model with the less Akaike information criterion (AIC). Explanatory variables were excluded when *p*‐value ≥0.05 was obtained. The predicted probabilities of a positive brucellosis herd ($\hat{p}$) were calculated from the log odds obtained in the generalized linear model, using the following formula (Bailey et al., 2002):

(5) $$\hat{p}=\frac{e^{\log\mathrm{odds}}}{\left(1+e^{\log\mathrm{odds}}\right)}\;.$$

The risk *P_R_* of brucellosis invasion from transhumance herds in host country (Côte d'Ivoire) is calculate with the following formula (Dean et al., 2013):

(6) $$P_{R}=\;1-{\left(1-p_{t}p_{\inf}\right)}^{n_{\mathrm{sold}}},$$

and

(7) $$p_{\inf}=\;1-1/R_{0}\;,\;$$

where *p_t_* is the probability to introduce an infected bovine in host regions (*p_t_* was assumed to correspond to brucellosis seroprevalence of herds in transhumance from Mali because of the low detection capacity of brucellosis at border control; Oyetola et al., 2021); *p*_inf_ is the probability of brucellosis outbreak in host regions in Côte d'Ivoire; *R*_0_ is the basic reproduction number or number of secondary cases resulting from the introduction of an infected bovine into a fully susceptible population; *n*_sold_ is the number of cattle sold from herds in transhumance from Mali in Côte d'Ivoire.

The risk *P_R_* was explored by varying *R*_0_ and using an estimate number of cattle sold during transhumance as transmission ways among infectious possible contacts. Introduction of infected bovine increased the risk of infection. The number of cattle sold was estimated with a rate of minimum and maximum annual cattle sales, and number of transhumant cattle during last transhumance in 2019.

### 2.6 Ethical considerations

Field activities received agreement with the national directions of veterinary health services of Côte d'Ivoire and Mali. The participation in this study was voluntary, breeders or herdsmen received detailing on the research objectives and procedures before agreeing to participate through giving written consent. Breeders or herdsmen of infected herds received advice from a veterinarian.

## 3 RESULTS

### 3.1 Brucellosis seroprevalence in cross‐border transhumant livestock

In transhumant cattle, the seroprevalences of brucellosis at individual and herd level were 3.6% (95% CI = 1.7–5.6) and 11.7% (95% CI = 4.6–18.8), respectively, and no positive animal was identified among 17 sheep from 4 of 5 mixed herds. Individual level seroprevalence was significantly different (*p*‐value = 0.0009) from one departure region in Mali to another one (Table 1). According to region of origin of herds, individual seroprevalence in cattle in transhumance from Sikasso was low compared to Segou (*p*‐value = 0.0383). In turn, seroprevalence in cattle in Segou was lower than in Koulikoro (*p*‐value = 0.0001). Brucellosis seroprevalence of transhumant cattle was not significantly different according to host region in Côte d'Ivoire.

**TABLE 1:** Individual and herd seroprevalence of brucellosis in transhumant cattle from Mali to Côte d'Ivoire.

	*n* Tested	Positive	Prev. in%	95% CI	*p*‐Value
Overall					
Individual	521	19	3.6	1.7–5.6	
Herd	111	13	11.7	4.6–18.8	
First transit region in Côte d'Ivoire (herds)			1 (0.371)		
Bagoue	304 (66)	11 (6)	3.6 (9.1)	1.1–6.1 (0.9–17.3)	
Folon	217 (45)	8 (7)	3.7 (15.6)	07–6.7 (3–28.1)	
Host regions in Côte d'Ivoire (herds)			0.182		
Bagoue	190 (40)	8 (4)	4.2 (10)	0.8–7.6 (0–21)	(0.353)
Bere	13 (3)	0	0	0	
Folon	59 (12)	3 (2)	5.1 (16.7)	0–11.7 (0–41.6)	
Kabadougou	185 (38)	5 (5)	2.7 (13.2)	0–5.5 (0.4–25.9)	
Poro	15 (3)	2 (1)	13.3 (33.3)	0–49.3 (0–96.5)	
Touba	12 (3)	1 (1)	8.3 (33.3)	0–26.8 (0–96.5)	
Worodougou	47 (12)	0	0	0	
Departure region in Mali (herds)			0.0009		
Koulikoro	45 (9)	7 (3)	15.6 (33.3)	3–28.1 (0–69.8)	(0.0501)
Mopti	10 (2)	0	0	0	
Segou	79 (16)	5 (4)	6.3 (25)	0–12.7 (0–50.1)	
Sikasso	382 (83)	7 (6)	1.8 (7.2)	0.2–3.4 (0.6–13.8)	
Tombouctou	5 (1)	0	0	0	
Herd composition (herds)					0.1235
Cattle	421 (90)	64 (13)	15.2 (14.4)	6.4–24 (5.9–23)	(0.1235)
Cattle and sheep	100 (21)	0	0	0	
Sex			0.7939		
Female	391	15	3.8	1.6–6.1	
Male	130	4	3.1	0–6.6	
Breed			0.1805		
*Bos taurus* (BT)	43	1	2.3	0–7.7	
*Bos indicus* (BI)	461	16	3.5	1.5‐5.4	
BT x BI	17	2	11.8	0–29.9	
Age categories			0.3117		
6 months−4 years	129	5	3.9	0–7.8	
5–8 years	260	12	4.6	1.6–7.6	
≥9 years	132	2	1.5	0–4	

*Note*: Values corresponding to herd level were indicated between brackets. Data were collected in herds from Mali to Côte d'Ivoire during the transhumance 2021.

Abbreviations: CI, Confidence interval; Prev., seroprevalence.

### 3.2 Seroprevalence of brucellosis in sedentary livestock

In sedentary cattle, the seroprevalence was 16.6% and 39.0% at individual and herd level, respectively. Sedentary cattle from the region of Segou were significantly more infected than in Sikasso at herd (72.0% vs. 14.7%) and individual (33.6% vs. 5.3%) level. Cattle from Segou had 8.9 (95% CI = 4.0–22.2) and 14.0 (95% CI = 3.5–67.3) more probability to be infected compared to those from Sikasso at individual and herd level, respectively. In Sikasso region, all infected sedentary cattle were in the Cercle of Kolondienba (Table 2).

**TABLE 2:** Individual and herd seroprevalence of brucellosis in sedentary cattle in Segou and Sikasso region, Mali.

	*n* Tested	Positive	Prev. in %	95% CI	*p*‐Value
Overall					
Individual	283	47	16.6	11.5–21.7	
Herd	59	23	39	24.3–53.7	
Region (herds)					5.2 × 10^−10^ (1.0 × 10^−5^)
Segou	113 (25)	38 (18)	33.6 (72)	23.3–43.9 (51.2–92.8)	
Sikasso	170 (34)	9 (5)	5.3 (14.7)	1.3–9.3 (0.6–28.8)	
Cercle Mali (herds)					8.1 × 10^−14^ (1.0 × 10^−5^)
Kadiolo	70 (14)	0	0	0	
Kolondienba	100 (20)	9 (5)	9 (25)	2.4–15.6 (2.5–47.5)	
Niono	113 (25)	38 (18)	33.6 (72)	23.3–43.9 (51.2–92.8)	
Breed					0.3693
*Bos taurus* (BT)	35	4	11.4	0–23.9	
*Bos indicus* (BI)	245	42	17.1	11.6–22.7	
BT × BI	3	1	33.3	0–96.5	
Sex					0.0756
Female	224	42	18.8	12.7–24.8	
Male	59	5	8.5	0.1–16.9	
Age categories					0.0893
≥6 months−4 years	118	13	11	4.3–17.7	
5−8 years	135	28	20.7	12.6–28.8	
≥9 years	30	6	20	3.1–36.9	

*Note*: Values corresponding to herd level were indicated between brackets. Data were collected in herds from Mali to Côte d'Ivoire during the transhumance 2021.

Abbreviations: CI, Confidence interval; Prev., seroprevalence.

### 3.3 Influence of mobility on brucellosis seroprevalence rate and infection

In cattle from Mali (sedentary and seasonal transhumant in Côte d'Ivoire), the overall seroprevalence was 8.2% (95% CI = 6.0–10.5) at the individual level and 21.2% (95% CI = 13.9–28.4) at the herd level. Seroprevalence in sedentary cattle was significantly higher than seroprevalence in transhumant cattle, both at the individual level (*p*‐value = 5.9 × 10^−10^) and at the herd level (*p*‐value = 6.2 × 10^−5^). The same observation was made in cattle native from the region of Segou (Table 3). In the Sikasso region, only the individual seroprevalence of sedentary cattle (5.3%) is higher than that of transhumant cattle (1.8%). Overall, cross‐border mobile herds had 0.2 less risk to have brucellosis infection compared to sedentary herds, and mobility was not a risk factor (95% CI = 0.08–0.48) of cattle brucellosis infection.

**TABLE 3:** Comparison of brucellosis seroprevalence of transhumant and sedentary cattle according to origin region in Mali.

	Transhumant		Sedentary				
	Pos.	Neg.	Pos.	Neg.	*p*‐Value	OR	CI_OR_
Herd level							
Segou region	4	12	18	7	0.0045	0.13	0.02–0.64
Sikasso region	6	77	5	29	0.2935	0.45	0.10–2.04
Overall^a^	13	98	23	36	6.2 × 10^−5^	0.20	0.08–0.48
Individual level							
Segou region	5	74	38	75	3.4 × 10^−6^	0.13	0.03–0.36
Sikasso region	7	375	9	161	0.0495	0.33	0.10–1.02
Overall^a^	19	502	47	236	5.9 × 10^−10^	0.19	0.10–0.33

Abbreviations: CI, confidence interval; OR, odds ratio; Pos., seropositive; Neg., seronegative.

^a^ Overall including all sample cases without restriction of region of origin of the herd.

### 3.4 Risks of cattle infection and transmission from cross‐border mobility

The only departure region which significantly impacts transhumant herd infection was Sikasso. Herds in transhumance from the region of Sikasso were 0.2 (95% CI = 0.058–0.923) less at risk to be infected by *Brucella* spp. than those from others regions. Previous brucellosis infection and unexplained abortion in herds in transhumance were risk factors of current herd infection (Table 4). Previous brucellosis infection of the herds was indicated by herdsmen who rightly described the disease or recognized the symptoms after disease description. Indeed, the ‘previous infection’ was strongly correlated to ‘unknown abortion aetiology’ observed in herds in transhumance with a kappa coefficient equal to 0.869 (95% CI = 0.757–0.981). Both factors were significant in a predictive model with the departure region singularly for Sikasso region. Although the model *∼Departure region + history of brucellosis* (AIC = 79.115) appeared sensibly better compared to the model *∼Departure region + unknown abortion aetiology* (AIC = 80.072), the second yielded better probability to predict a positive herd from Sikasso (Table 5).

**TABLE 4:** Factor influencing brucellosis infection in transhumant herds.

Studied factor	*n* Herd	Prev. in %	*p*‐Value	OR	95% CI_OR_
Origin (Sikasso)	83	7.2	0.0183	0.237	0.058–0.923
Previous infection	84	92.9	0.0146	4.471	1.146–18.118
Unknown abortion	22	27.3	0.0209	4.314	1.051–17.360

*Note*: A total of 111 herds in transhumance were included in the analysis and previous infected herds were designate by herdsmen themselves according to their knowledges on brucellosis signs.

Abbreviations: CI, confidence interval; OR, odds ratio; Prev., seroprevalence.

**TABLE 5:** Prediction of the probability of positive Rose Bengal test (RBT) result for a herd in transhumance from Mali.

	Estimate	95% CI_OR_	*p*‐Value	Charact.	Log odds component
Model 1: *RBT – departure region + history of brucellosis*					
Intercept	−1.0801				−1.0801
Departure region (koulikoro vs.)					
Mopti	−17.4348	0‐inf	0.9948	No	0
Segou	−0.8371	0.06–3.03	0.3988	No	0
Sikasso	−1.9553	0.02–0.78	0.0248	No	0
Tombouctou	−18.0252	0‐inf	0.9964	No	0
History of brucellosis					
Infection no vs. yes	1.5393	1.28–17	0.0197	Yes	1.5393
Model 2: *RBT – departure region + unexplained abortion*					
Intercept	−1.0548				−1.0548
Departure region (koulikoro vs.)					
Mopti	−17.3871	0‐inf	0.9950	No	0
Segou	−0.7040	0.07–3.34	0.4700	No	0
Sikasso	−1.8397	0.02–0.85	0.0322	No	0
Tombouctou	−17.9545	0‐inf	0.9960	No	0
Signs of brucellosis					
Abortion no vs. yes	1.4432	1.14–15.7	0.0307	Yes	1.4432

*Note*: CI_OR_ is the confidence interval of odds ratio; inf is the infinity. Charact. is the characteristic, this indicated component use for the sum of the log odds component are 0.4592 and 0.3884 for Models 1 and 2, respectively. The predicted probabilities of a positive brucellosis herd were 0.612 and 0.595 for Models 1 and 2, respectively. These probabilities decreased, if the herd coming from Sikasso and become better in model 2 (0.189) compare to model 1 (0.183).

**FIGURE 2:**
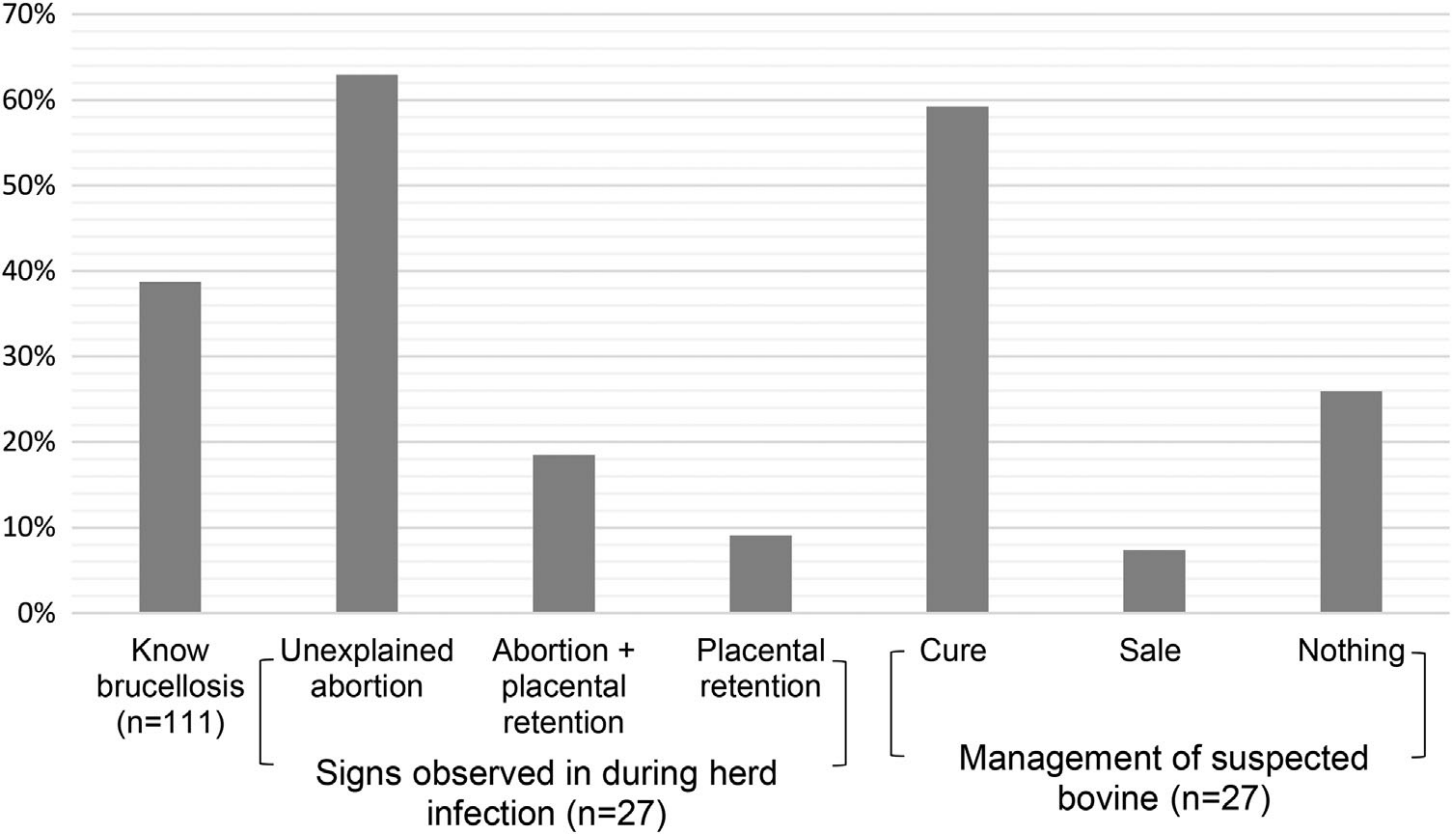
Proportion of cross border pastoralists who knew brucellosis and related signs occurred during herd infection. Brucellosis was knowing in Fulani language as Bakalé (26/111) and in Senufo language as *konofilibana* (17/111). Signs observed and management of suspicion cattle were indicated by herdsmen (27/111) who self‐think that their herd was infected.

**FIGURE 3:**
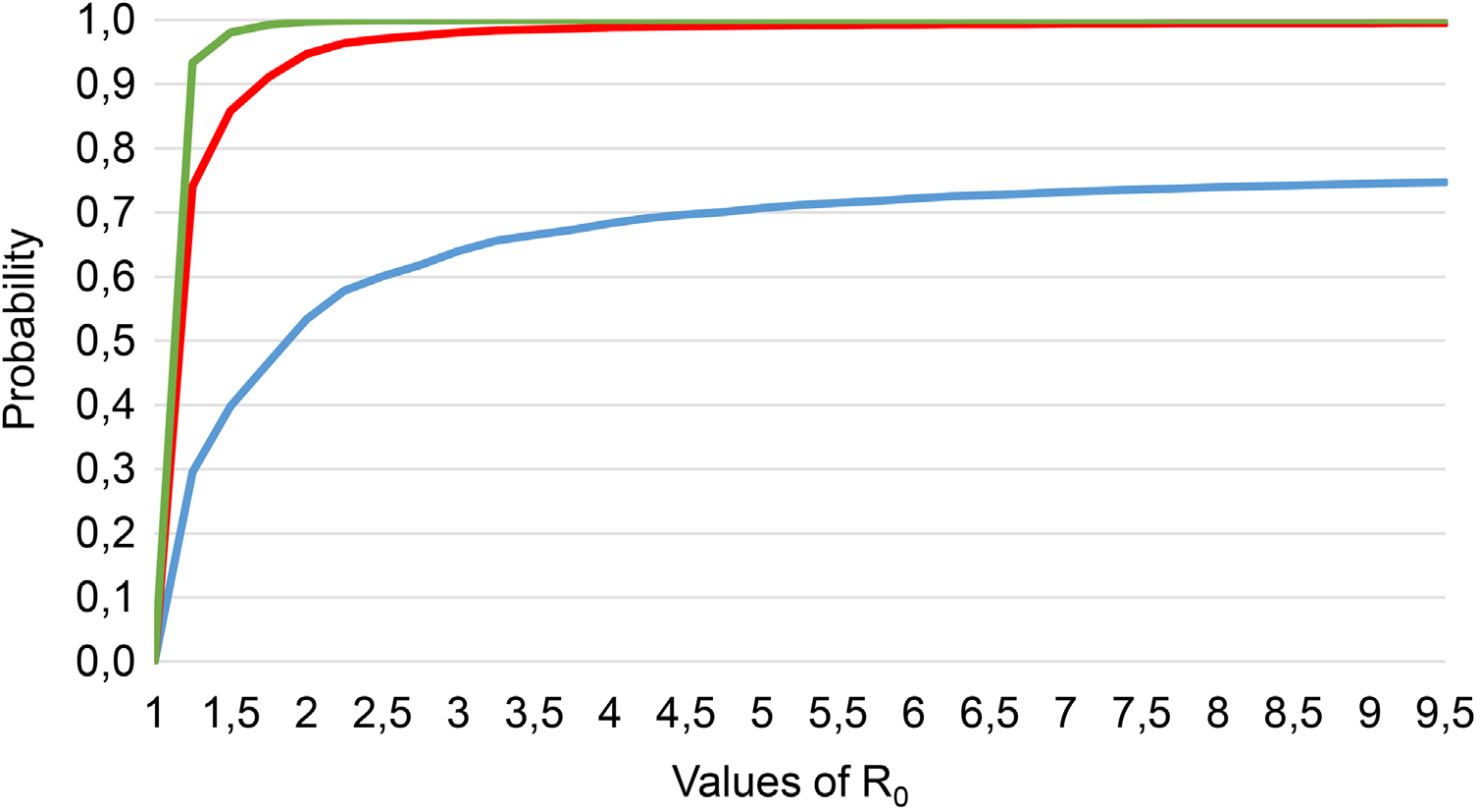
Probability of brucellosis invasion in cattle population of host regions (Bagoue and Folon regions) in Côte d'Ivoire through cattle sold from herds in transhumance from Mali. Line blue is the variation of invasion probability considering the number of cattle sold (42) from the sample herds. Overall, 0.3% to 0.6% of cattle would be sold during the annual transhumance in Bagoue and Folon regions. Respectively, lines green and red correspond to invasion probabilities from maximum (216) and minimum (108) estimated number of cattle sold among 54,139 cattle in transhumance in 2019 from Mali.

Overall, 24% of the 111 cross‐border pastoralists interviewed had indicated that their herd had a history of brucellosis infection, based on their own knowledge on the disease or the description provided by the investigator during interviews. Observed signs were abortion and placental retention. In case of suspicion of cattle brucellosis infection, 59% and 7.4% from herdsmen applied a self‐treatment for cattle using antibiotics and sold the suspected cattle, respectively (Figure 2). Overall, 48.6% (95% CI = 37.6–59.7) of transhumant pastoralists sold on average two cattle or 2.5% of their cattle, mainly to meet unforeseen expenses. In total, we estimated that sale concerned 0.3%–0.6% of cattle in transhumance. With a *R*_0_ around 1.25, the risk of *Brucella* spread was between 0.7 and 0.9, from transhumant cattle sold in host regions in Côte d'Ivoire (Figure 3).

## 4 DISCUSSION

This study provides recent data on bovine brucellosis in Mali with a focus on mixed mobile and sedentary livestock systems by exploring the risks of contamination and spread due to cross‐border mobility. Indeed, although the overall seroprevalence was 21.2% at herd level in cattle from Mali, cross‐border mobile herds were less infected than sedentary herds. Transhumant herds with unknown abortion aetiology indicated *Brucella* spp. infection with a probability of 61% according to the predictive model designed, and cattle sale constituted a high‐risk point of brucellosis spread in the visited areas.

The overall herd seroprevalence of brucellosis obtained in this study is low compared to that published by Tounkara et al. (1994) on sera collected during the national surveillance programme in 1991 (53% ± 6.4%). The individual seroprevalence of brucellosis obtained in this study is lower than what was observed in 1991 (23.3%; Tounkara et al., 1994) and 2013 (19.7%; Cissé, 2015) in Mali and about equal to the 11.6% positive samples among 626 blood samples from Bamako and Koulikoro regions, tested with RBT by Laboratoire Central Vétérinaire (LCV) in 2020 (Laboratoire Central Vétérinaire [LCV], 2021). This significant reduction of brucellosis in Mali could be linked to livestock mobility and loss of chronic infected cattle led by droughts, other diseases and trading for meat. Indeed, as shown in the results of this study, transhumant herds (11.7%) were significantly less infected by brucellosis compared to sedentary herds (39%). This result is contrary to that observed in Niger, where the risk of seropositivity was more important for herds practicing transhumance than sedentary ones (Boukary et al., 2013). Sedentary herds which do not leave their region but are in contact with other herds, including those going on transhumance as in this study, could be contaminated, and inter‐herds promiscuity could increase infection in sedentary herds. In addition, in sedentary livestock, some livestock management like joint herds for grazing because of herdsmen shortage, and exchange of bulls promoted inter‐herds contact. Transhumance promotes long distance and rapid spread of diseases (Apolloni et al., 2019; Oyetola et al., 2021). However, transhumance appears as a mitigating factor of *Brucella* exposure, compared to sedentary farming which is associated with intensification of livestock density, particularly in peri‐urban areas (Ducrotoy et al., 2017; Musallam et al., 2019).

Sedentary cattle from the region of Segou were significantly more infected than those from Sikasso. In 1991, there was an inverse situation observed in both regions, with a seroprevalence of 66% vs. 44% at herd level and 26.4% vs. 13.6% at individual level. During that time, livestock was only sedentary in Sikasso and sedentary and transhumant in Segou (Tounkara et al., 1994). Livestock system adaptation in the region of Sikasso due to droughts, pressure of herd coming from Sahelian regions in the north of Mali and reduction of grazing area led to cross‐border mobility of the herd and could probably have mitigated brucellosis exposure. However, the intensification of livestock in peri‐urban zones for dairy production in the region of Segou facilitated the increase of disease. The peri‐urban zone of Bamako presented a high brucellosis seroprevalence (32.5%) in dairy herds (Musallam et al., 2019), reinforcing the assertion that this type of livestock system as practiced promotes brucellosis infection and increase circulation of pathogens. Indeed, local selections of cows and bulls are not made considering their brucellosis status, but dairy performance and tolerance of trypanosomiasis (Ouédraogo et al., 2021) and their admission into a herd constitute a risk of disease introduction.

In transhumant livestock, significant variation of individual seropositivity to brucellosis according to their departure region in Mali and risk factors of infection such as unknown abortion aetiology could be used to implement control test based on risk in border mechanism control. This control strategy could help to save money and time during border inspection. In addition to contact with transhumant herds (Kanouté et al., 2017), another more permanent factor as the sale of cattle from transhumant herds would be involved in the risk level of spread of brucellosis. The risk of brucellosis spread from cattle sale during transhumance appeared high (70%–90%) with a prevalence of 3.6% and assuming a *R*_0_ around 1.25. The probability of disease introduction from the market system in Togo (80%) is similar to our estimation, with a same value of *R*_0_ and a prevalence of less than 1% (Dean et al., 2013). The risk could be less influenced by cross‐border transhumance than cross‐border trade, probably because the main objective during transhumance was access to pasture and water and not cattle trade. Selling of cattle in transhumance was less in this study than the rate observed (around 5%) in other transhumance axes (Thebaud, 2017). One explanation could be that the transportation of livestock on foot for trade is prohibited since 2016 in Côte d'Ivoire (République de Côte d'Ivoire, 2016). However, some pastoralists must sell their cattle to local herders to pay the fines for crop damages.

Sheep tested from mixed transhumant herds did not have antibodies against *Brucella* spp. even though some studies show an individual seroprevalence of 4.6% (95%CI = 1.5–5.6) in the circle of Bamako, Segou and Sikasso (Traoré et al., 2021) and from 1% (Sow, 2011) to 37.1% (Coulibaly, 2016) according to the circle in the region of Segou, in Mali.

Limitations of this study were the area explored for sedentary livestock, although the two main regions of departure (Sikasso and Segou) were explored, and data from the third region (Koulikoro) are available from LCV without being published. The sampling of the study could be improved by considering the region as the sampling stage; the number of herds per region could be estimated according to their distribution in each region for sedentary or entry region for transhumance. However, the proportion of transhumant herds sampled per entry region (Folon and Bagoue) was almost similar to the frequency of cattle per region recorded at the border during the last transhumance. Low number of sheep tested from a mixed herd constituted a limit to explore his impact on herd seropositivity to *Brucella* spp., whereas sheep are associated to cattle herd infection (Ducrotoy et al., 2017; Kanouté et al., 2017). Another limitation was the using of only RBT for diagnostic. This test RBT gives false positive resulting to the same antigenic characters between of similitude of *Brucella* spp. and others pathogens like *Yersinia* and *Salmonella* (Maurin, 2005), and false negative during the incubation period. However, its sensitivity and specificity at herd level make it suitable for herd screening of brucellosis. RBT is recommended by the World Organisation for Animal Health (WOAH, founded as OIE) for herd prevalence of infection (World Organisation for Animal Health [WOAH], 2022). The risk of spread estimated did not take into account the current infection existing in the host regions, which is around 4.6% in northern Côte d'Ivoire (Kanouté et al., 2017). Furthermore, we assumed that the animals sold were incorporated into local herds, as the systematic slaughter after purchase seems less obvious but not impossible. The destination/future of the animals sold need to be investigated to separate the risk linked to human exposure (butchers, veterinarians, consumers …) and local herds.

## 5 CONCLUSION

In conclusion, this study highlights that cross‐border transhumance has a complex role in disease dynamic in cattle population at regional scale, although it probably reduces exposure to some diseases as brucellosis. Risk‐based border controls require a full captured of cross‐border pastoralists through border control and testing of marketed animal. To mitigate risk of brucellosis spread through transhumance, it appears important to understand situations leading to sale of cattle during transhumance and address control of this trade.

## AUTHOR CONTRIBUTIONS

*Conceptualization; data curation; formal analysis; investigation; methodology; resources; software; validation; visualization; writing – original draft preparation; writing – review and editing*: Wilfried Délé Oyetola*. Investigation; data curation; formal analysis; visualization; writing – review and editing*: Samba daou*. conceptualization; funding acquisition; project administration; methodology; supervision; validation; writing – review and editing*: Bassirou Bonfoh*. Conceptualization; methodology; supervision; validation; writing – review and editing*: Rianatou Bada Alambedji.

## CONFLICT OF INTEREST STATEMENT

The authors declare that the research was conducted in the absence of any commercial or financial relationships that could be construed as potential conflicts of interest.

## ETHICS STATEMENT

The authors confirm that the ethical policies of the journal, as noted on the journal's author guidelines page, have been adhered to and the appropriate ethical review committee approval has been received. This study involving animal sampling was reviewed and approved by two ethics committee: ‘Comité d'Ethique de la Recherche de l'Université Cheikh Anta Diop de Dakar – Sénégal’ with reference Protocole0317/2018/CER/UCAD and ‘Comité National d'Ethique des Sciences de la Vie et de la Santé (CNESVS) du Ministère de la Santé et de l'Hygiène Publique of Côte d'Ivoire’ with reference 142‐18/MSHP/CNESVS‐km.

### PEER REVIEW

The peer review history for this article is available at https://publons.com/publon/10.1002/vms3.1446.

## Data Availability

The data that support the findings of this study are available from the corresponding author upon reasonable request.
